# Groundnut Production and Storage in the Sahel: Challenges and Opportunities in the Maradi and Zinder Regions of Niger

**DOI:** 10.5539/jas.v11n4p25

**Published:** 2019-03-15

**Authors:** Ousmane Bakoye, Ibrahim Baoua, Lawali Sitou, Mahamane Rabé Moctar, Laouali Amadou, Anastasia W. Njoroge, Larry L. Murdock, Dieudonne Baributsa

**Affiliations:** 1 Institut National de la Recherche Agronomique du Niger, Maradi, Niger; 2 Université DanDicko DanKoulodo de Maradi, Maradi, Niger; 3 Purdue University, West Lafayette, IN, USA

**Keywords:** groundnut value chain, storage pests, Sahel, marketing, processing

## Abstract

Groundnut *Arachis hypogaea* (L.), is an important legume crop after cowpea *Vigna unguiculata* (L. Walp) in Niger. However, there has been a decline in its economic importance due to several challenges. A survey of 800 farmers was conducted in 40 villages in the Maradi and Zinder regions to assess constraints and opportunities to improve groundnut production and marketing. Average land size and yield varied by region: 1.3 ha per farmer and 461.3 kg ha^-1^ in Maradi, and 1.7 ha per farmer and 417.2 kg ha^-1^ in Zinder. Insect pests (aphids) were the most important production constraint. Groundnut is typically stored for six to eight months after harvest but 91% of farmers do not take any precautions to protect the grain. Storage enables farmers to earn high profit margins of up to 33 and 113% for unshelled and shelled groundnuts, respectively. Most farmers (71.5%) sell their groundnut in unshelled form in local and urban markets. Traders are the main buyers according to 61.7% of farmers while processors were mentioned as purchasers by less than 20%. Sales are mostly done by individual farmers while very little is sold through cooperatives. Given that groundnut is a profitable crop adapted to the Sahelian zone, there is need to improve its production, storage, and value addition through processing.

The research is supported by Purdue University through the PICS3 project funded by the Bill and Melinda Gates Foundation.

## 1. Introduction

*Arachis hypogaea* (L.), commonly known as groundnut or peanut, is an important oil, food and feed legume crop originally from South America (Hammons, 1994). It is a valuable food crop because of its high oil content (43-55%) and protein content (25-28%), and provides vitamins and minerals for millions of households (Reddy et al., 2003). In addition, after oil extraction, the residual groundnut cake is processed into animal feed. Groundnut is now widely grown throughout the tropical, sub-tropical and warm temperate areas in Asia, Africa, Oceania, North and South America, and Europe (Freeman et al., 1999). Groundnut was introduced to West Africa by Portuguese explorers in the 16^th^ century (Hammons, 1973). Niger, which produces about 453,577 tons per year (FAO, 2016), is ranked 7^th^ among the major groundnut producers in Africa after Nigeria, Sudan, Chad, Cameroon, Senegal, and Tanzania. However, in 2016, groundnut productivity in Niger (588.2 kg ha^-1^) was low compared to leading producers in West Africa, *e.g.*, Nigeria (1,130 kg ha^-1^) and Senegal (817 kg ha^-1^) (FAOSTAT, 2016).

In Niger, groundnut is the second most important legume crop after cowpea (*Vigna unguiculata* L. Walp) (Hampson et al., 2001). Thus, groundnut in Niger plays a major role in local, regional and international/export markets. Exports from Niger to Europe began in 1885 with nearly two-thirds of Nigerien farmers producing groundnut in the 1930s thanks to the incentive prices offered by European markets (Rashkov, 2001). Based on FAO (2016), Niger’s production of unshelled nuts has increased from 147,000 MT in 2007 to more than 450,000 MT in 2016. Most of this increase in yield is due to adding more land into groundnut production together with limited yield increases. Niger’s groundnut production and yields have been affected over the years by low performing varieties, drought, pest attacks and low prices linked to the entry of soybeans into the world market of oilseed crops (Ntare et al., 2004; Ndjeunga et al., 2008; Coulibaly et al., 2017). A large proportion of the Niger production (75 %) comes from the regions of Maradi and Zinder (M.A., 2018).

Field pests are one of the major challenges affecting groundnut production. There are several species of groundnut field insect pests which are responsible for substantial yield losses (Biswas, 2014). Among these pests are aphids that are vectors of the rosette which is the most destructive virus disease of groundnut (Naidu et al., 1998; Waliyar et al., 2007). In addition to preharvest contraints, there are several challenges associated with groundnut postharvest management practices. These challenges are linked to poor drying and storage that result in microbial contamination, and pest (insect, rodent, *etc*.) attacks. Postharvest losses due to pests and poor management practices can reach up to 70% after six months of storage (Oaya et al., 2012). Other groundnut postharvest challenges include grain biochemical changes (flavor change, rancidity, viability loss), physical changes (shrinkage and weight loss), and absorption of odors and chemicals during storage. Poor postharvest management practices not only increase losses, but also reduce the quality and value of the groundnuts and hence access to market. Baidu-Forson et al. (1997) noted that farmers’ access to markets for unshelled groundnut was an important challenge that required intervention in Niger.

In view of these pre- and post-harvest challenges to developing the groundnut value chain in Niger, a study was conducted to assess farmers’ constraints and opportunities to improve production and marketing. Increased groundnut production and access to markets will increase food security and income of farmers in Niger. Our main objectives were to: (i) assess quantity of groundnut produced and stored; (ii) evaluate constraints during production and storage, and (iii) learn more about marketing and sale of groundnuts.

## 2. Methods

### 2.1 Study Sites

This survey was implemented from 19 to 29 March 2016 in two regions of Niger (Maradi and Zinder). With approximately 75% of the national production (M.A., 2018), these two regions are the largest producers of groundnut in Niger. Maradi and Zinder regions are located in south-central Niger and have soils that are predominantly sandy. The annual rainfall in these regions varies from 400 to 500 mm. The survey was conducted in four departments (districts): Guidan Roumdji and Madarounfa in Maradi region and, Mirriah and Kantché in Zinder region.

### 2.2 Sampling and Data Collection

Data was collected from 40 villages in the four departments (Table 1). The villages were randomly selected from a list of villages in each department. The number of villages assigned to each department was determined based on the average production of unshelled groundnut in each department over the last five years. In each village, 20 groundnut producers were randomly selected during a public meeting resulting in a total of 800 farmers.

**Table 1:** Average annual production of groundnuts by department, and number of villages and households surveyed in the Maradi and Zinder regions of Niger

Regions	Departments	Average production of the last 5 years in tons	# of villages	# of households
Maradi	Madarounfa	54 340	13	260
	Guidan-Roumdji	45 144	11	220
Zinder	Kantché	43 492	10	200
	Mirriah	23 183	6	120
Total		166 159	40	800

A questionnaire consisting of open and closed-ended questions was developed and uploaded into the Open Data Kit (ODK) server and then downloaded on tablets. The tablets were used by enumerators to collect data from each respondent. The data was saved on the tablets after each interview and uploaded to the ODK server daily when internet access was available. Key questions provided information on: 1) respondant demographics; 2) groundnut production, quantity stored and location of storage; 3) main challenges during production and storage; 4) groundnut marketing; 5) price data during harvest and during the lean season.

### 2.3 Data Analysis

Data was downloaded from the ODK server as an excel file, cleaned, and transferred to SPSS Version 16 (SPSS Inc. 2007) for statistical analysis. Descriptive statistics and frequencies were used to analyze the data. ANOVA tests followed by LSD were used to compare prices between different periods of the year and the averages of the variables on production across departments.

## 3. Results

The demographic data are presented in Table 2. The average age of the respondents was 48 years with experience growing groundnuts ranging from 20 to 25 years. About 22% of the respondents were women with a range of 14 to 26% among the four departments. Most of the respondents (89.6%) were engaged in agriculture and a few of them (4.4%) were involved in trade. More than half of the respondents were literate (57%) having attended either Koranic or formal schools. On average, the area cultivated by farmers was 1.3±0.3 ha in the Maradi region and 1.7±0.2 ha in Zinder (data not shown). Average yields varied by the department with 464.6 kg ha^-1^ in Guidan Roumdji, 456.5 kg ha^-1^ in Madarounfa, 476.7 kg ha^-1^ in Kantché, and 360.4 kg ha^-1^ in Mirriah (data not shown). Average yield per region was 461.3 kg ha^-1^ in Maradi, and 417.2 kg ha^-1^ in Zinder (data not shown). A little over half of the producers (54.9%) used local varieties.

**Table 2:** Average age and main activities of respondents in four departments in the Maradi and Zinder regions of Niger

Departments	Average age	% women	# of years growing groundnut	Main activities (% of respondents)			
				Agriculture	Livestock	Trade	Others
Madarounfa	48.5±0.9	28.0	19.8±1.1	85.8	0.8	4.6	8.8
Guidan Roumdji	49.8±0.9	25.5	25.0 ±1.2	92.1	-	4.7	3.3
Kantché	47.2±1.0	16.7	21.1±0.9	92.0	-	2.5	5.5
Mirriah	47.5±1.2	14.3	20.3±1.2	88.5	-	7.1	4.4
Overall Mean	48.4±0.5	22.4	21.6 ±0.6	89.6	0.3	4.4	5.6

The important groundnut production constraints are shown in Table 3. Insects, particularly aphids, were considered the main groundnut production constraint by 87.5% of respondents. Drought was the second most often cited constraint (11%), followed by varietal degeneration (6.7%) and low soil fertility (6.4%).

**Table 3:** Main constraints of groundnut production in four departments in the Maradi and Zinder regions of Niger

Department			Constraint (% of respondents)		
	Insects	Diseases	Drought	Varietal degeneration	Low soil fertility
Madarounfa	89.2	1.9	3.10	8.1	3.9
Guidan Roumdji	88.8	4.3	12.87	3.4	2.6
Kantché	85.6	5.8	22.11	10.1	13.5
Mirriah	84.9	0.0	5.04	2.5	6.7
Overall Mean	87.5	3.3	11	6.7	6.4

Among the 4 departments, 89 to 96% of producers reported that they stored groundnuts they had produced (Table 4). Quantities stored varied from 5 to 14 kg for shelled groundnuts and from 165 to 243 kg for unshelled groundnuts, depending on the locality. Unshelled groundnuts contributed from 94 to 100% of the inventories. The main reasons for storage were to generate income at a later date, and seed preservation. Less than 1% of respondents stored groundnuts for home consumption. Storage was carried out in the family home by 92 to 97% of producers; while less than 2% used granaries made out of straw or clay, and less than 3% used cooperative stores in the village (data not shown).

**Table 4:** Proportion of producers storing groundnuts, average quantity stored and purpose of storage, in four departments in the Maradi and Zinder regions of Niger

Departments	% Production stored	Quantity of groundnut stored (kg)		Purpose of storage		
		Shelled	Unshelled	Income	Seed	Consumption
Madarounfa	93.3±1.4	14.0±8.4	220.7±28.6	62.4%	37.6%	-
Guidan-Roumdji	89.4±2.0	7.5±6.5	243.4±34.9	45.1%	54.9%	-
Kantché	96.2±1.1	4.6±2.8	165.0±28.0	46.1%	53.3%	0.6%
Mirriah	91.3±2.0	0.00	182.2±33.3	61.4%	36.4%	2.3%
Overall Mean	92.7±0.8	7.7±3.3	208.2±16.1	53.1%	46.5%	0.4%

Several insect pest species attack groundnuts during storage (Table 5). *Corcyra cephalonica* (Stainton, 1866) was mentioned by 64.9% of the respondents as the main insect pest of stored groundnut while *Caryedon serratus* (Olivier, 1790) was mentioned by less than 20% of them. The flour worm *Tribolium* sp. was reported by less than 15% of producers. Other species such as *Cryptolestes* sp. and *Trogoderma granarium* (Everts, 1898) were also reported by 2 to 10% of respondents. Pest control methods were rare; some 88 to 94% of the repondents took no steps to preserve their grains, while the rest reported fumigating with phostoxin or using contact insecticides. The use of hermetic technologies including the Purdue Improved Crop Storage (PICS) bag (Baributsa et al., 2017) was not mentioned by any of the respondents.

**Table 5:** Proportion of farmers’ responses regarding major insect pests of groundnut stores in four departments in the Maradi and Zinder regions of Niger

Departments	Insect species (% of respondents)			
	*C. serratus*	*C. cephalonica*	*Tribolium* sp.	Others
Madarounfa	33.6	51.7	12.1	2.6
Guidan Roumdji	14.6	62.9	13.5	9.0
Kantché	6.9	73.3	9.5	10.3
Mirriah	0	79.6	18.4	2.0
Overall mean	16.2	64.9	12.4	6.5

In the departments of Guidan-Roumdji, Madarounfa and Mirriah, most of the respondents (74 to 98%) sold their groundnuts in shelled form (Table 6). By contrast, in the department of Kantché, most respondents (80%) sold their groundnuts in unshelled form. A majority of farmers (64.9%) sold their groundnuts into urban and rural markets. About a third of them (32.9%) sold their groundnuts from home. Selling through farmers’ organizations was mentioned by less than 3% of the respondents. The majority of respondents stated that the major groundnut buyers were traders (79.2 %), followed by processors (18.6%) (Table 6). Processors were described as the women who extracted and retailed groundnut oil and oil cake. Farmers who bought groundnut accounted for only about 2.2% of the customers. These farmers were mostly purchasing seeds to plant.

**Table 6:** Characteristics of the groundnut sales network in four departments in the Maradi and Zinder regions of Niger

Departments	Type of groundnut sold		Point of sale			Buyers		
	Shelled	Unshelled	Market	Farmers’ organizations	Sales at home	Traders	Processors	Farmers
Madarounfa	8.8	91.2	60.5	3.9	25.6	68.6	28.0	3.4
Guidan-Roumdji	1.8	98.2	50.0	1.6	48.4	92.8	5.6	1.7
Kantché	80.0	20.0	83.9	1.6	14.4	83.6	15.3	1.1
Mirriah	26.4	73.6	45.1	2.2	52.7	63.2	33.3	3.4
Overall mean	28.5	71.5	64.9	2.3	32.8	79.2	18.6	2.2

**Figure 1:**
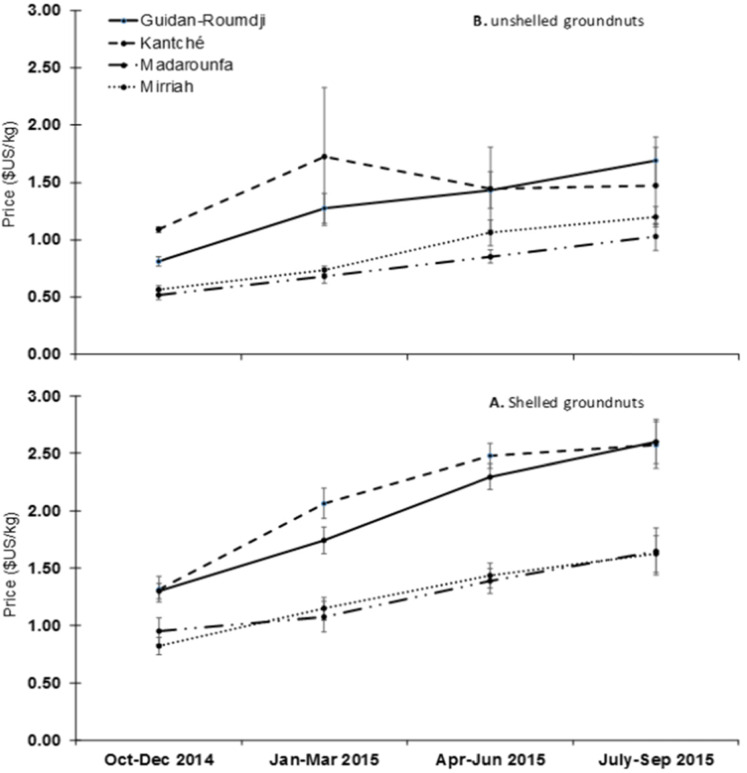
Seasonal evolution of the price per kilogram for shelled (A) and unshelled (B) groundnut in Maradi and Zinder regions of Niger

The seasonal variation in the price of shelled and unshelled groundnuts is shown in Figure 1. The average price of groundnuts in the four departments varied throughout the year. The average prices for shelled groundnuts varied between a low of $1.1/kg in the period of harvest time (October-December 2014) to a high of $2.11 during the lean season (July-September 2015) when supply is low (Figure 1A). For each season, the prices recorded in the departments of Guidan-Roumdji and Kantché were higher compared to those reported in the other entities with differences varying between 36 to 92%.

The average price increase for unshelled groundnuts followed the same trend as with shelled groundnuts. The average price of unshelled groundnuts in the four departments varied from season to season. It varied from $0.74/kg from harvest (October to December 2014) to $1.35/kg during the lean season (July to September 2015) (Figure 1B). Similar to shelled groundnuts, the prices of unshelled groundnuts recorded in the departments of Guidan-Roumdji and Kantché were higher than those of the departments of Madarounfa and Mirriah, with differences varying between 22 to 152%. It is interesting to note that the average price of unshelled groundnuts in the department of Kantché did not differ throughout the year (F = 0.50, df = 3/35, p = 0.67).

## 4. Discussion

Groundnuts are a significant source of income for West African farmers but this legume has experienced a significant decline in export over the last 30 years (Noba et al., 2014). Groundnuts are an important source of revenue for women who constitute a significant segment of the rural population. The present survey was conducted in areas where 75% of Nigerien groundnut is produced. The annual production of 400,000 tons in 2016 is not far above the 355,000 tons produced in 1966, 50 years ago. Groundnuts occupy an average of 1.3 ha with a production of 351kg ha^-1^ despite its yield potential of 1.5 to 2 tons ha^-1^ (Hamidou et al., 2018). The average yield of 445.6 kg ha^-1^ for both regions was higher than the 351kg ha^-1^ reported by Hamidou et al. (2018) but lower than the 588.2 kg ha^-1^ reported by FAO (2016). These numbers point to a need for intervention, as current groundut yields in both regions are about a quarter of their potential.

As with several other studies, farmers mentioned several biotic and abiotic constraints that lead to low groundnuts yields. These include insect pests such as aphids that transmit diseases, drought due to poor distribution of rains, and low performing varieties due to varietal degeneration and limited access to quality seeds and new varieties (Busolo-Bulafu, 2004; Okello et al., 2010; Clavel et al., 2005; Rajitha et al., 2018). The respondents knew only one variety, 55-437, developed in 1955 (FAO, 1955). Yet, in 2003, at least 17 new high-performing varieties had been demonstrated at different sites in the country (Ndjeunga et al., 2008). This shows that there has been little change in groundnut production, especially in the adoption of improved varieties, despite advances in breeding of new planting materials. Therefore, greater efforts need to be made to disseminate new varieties and to improve their access for farmers. Low soil fertility for groundnut production, which is now a recurrent problem in the Sahel, was also reported in Senegal (Pessis, 2013). Integrated soil fertility management techniques using locally available resources have been developed (Ouédraogo et al., 2001; Vanlauwe, 2010; Hamidou, 2018; Kadanga & Sogbedji, 2017). Updating extension programs of local public agricultural services should also be considered to build awareness among producers to improve soil management for increasing yields.

Most of the groundnut production is sold, very little (less than 1% on average) is consumed locally. Between 89% and 96% of the groundnuts produced are stored, certainly due to the lack of better prices after harvest in local markets as has been noted in Ghana and Senegal (Noba et al., 2014; Owusu-Adjei et al., 2017). Groundnuts are mostly stored unshelled, to better protect them from insect pest attacks. It is well known that pods are less susceptible to insect pest attacks compared to grains (Rao et al. 2010; Baributsa et al., 2017). During storage, which lasts 6 to 8 months, several insects have been recorded, mainly Lepidoptera *C. cephalonica*, which is well known as a groundnut pest (Senguttuvan et al., 1995; Baoua et al., 2015). *Caryedon serratus*, considered as one of the major groundnut pests (Rao et al., 2010; Guèye et al., 2011), was mentioned by less than 20% of respondents. For postharvest control methods, 88 to 94% of the respondents do not take any action to preserve the grains against these storage pests. Only a small percentage of farmers use pesticides. The non-use of insect control methods by a large majority of farmers may be related to very low weight losses of stored unshelled groundnuts associated with insect pests. Estimated storage weight losses in Niger ranged from 8 to 14% after 6 to 7 months of storage (Baoua et al., 2015; Baributsa et al., 2017) and this ratio is quite low compared to that recorded on cowpea, which varies from 66 to 70% after 5 months of storage (Baoua et al., 2012). In Nigeria, weight losses of groundnuts are even higher and estimated to be around 70% and 68% for shelled and unshelled groundnuts, respectively (Oaya et al., 2012).

It should also be noted that the incidence of insect pests is not limited to physical damage to the grains. Groundnut is also subject to contamination by aflatoxin, a toxic and carcinogenic substance whose presence is explained by poor postharvest management practices (Anderson et al., 1995; Craufurd et al., 2006). The presence of insect pests and their movements within the store favors the contamination of the stored grain by this toxin (Hell et al., 2000) and is another reason why it is important to take measures to preserve groundnut during storage. Exploring the use of chemical-free hermetic technologies such as the PICS bags, that are commercially available in Niger, is important. PICS bags have shown to be effective for the preservation of groundnuts against the insect attacks and aflatoxin mitigation (Sudini et al., 2015; Baributsa et al., 2017).

The price per kilogram of groundnuts increased by 92.8% and 82.1% from harvest to the lean season for shelled and unshelled groundnuts, respectively. After taking into account the dehulling ratio of 2/3 and labor costs, the profit margins of shelled and unshelled groundnuts are comparable. The average prices of grounduts in Niger in January-March 2015 were $1.1/kg for unshelled and $1.51/kg for shelled. Looking at the price trend during the same period, these prices were significantly higher than those recorded in Senegal in February 2017, namely $0.41 for unshelled groundnuts and $0.95 for shelled groundnuts (SIM-Senegal, 2017). Price variations between departments during the same period depended on the production levels and access to markets or buyers. Prices are determined by supply and demand thus, the markets of Kantché and Guidan Roumdji can be considered as the main collection centers for groundnuts. Supplies are sold after 6 to 8 months of storage with gross profit margins of 73 to 101% for shelled groundnuts and 35 to 113% for in-shell groundnuts. These margins are higher than those realized by cowpea farmers in West Africa, which varies from 25 to 55% after 6 months of storage (Baributsa et al., 2014). Groundnut farming in Niger is a profitable activity because the demand is greater than the national production. By storing groundnuts for several months, farmers can even earn more money as prices increase from harvest into the lean season when supply is low.

Most farmers (89%) sold their stored groundnuts to traders including middlemen who buy from local markets and villages. Most of this groundnut is probably exported to Nigeria due to the proximity of these districts to the border (Baidu-Forson, 1997; Hamadou et al., 2000). As in Senegal and other countries in the region, groundnut production in Niger was fuelled by export markets to Europe before it collapsed in the 1980’s due to issues of grain quality and food safety related to aflatoxin contamination (Bonnefond & Couty, 1988; Ndiaye et al., 2016). Groundnut buyers who process the commodity accounted for less than 20% of farmers’ customers. With more

than 71.5% of groundnut sold in unshelled form and with no value addition, farmers are missing the opportunity to generate additional income. A study conducted in Ghana showed that when a farmer sells groundnut in unshelled form, most of the profits go to distributors (Owusu-Adjei et al., 2017). Thus, it is important that farmers use hermetic bags to cost-effectively store shelled groundnuts to increase their revenues (Baributsa et al., 2017). More than 46% of groundnut producers also sold some of their groundnuts as seed. Even if producers make up only 2% of buyers, this points to the existence of an informal seed system and perhaps explains the degeneration of varieties. Those respondents who sold groundnuts seed more likely saved some for planting. It is also necessary that extension services and other actors in the groundnut value chain make efforts to disseminate and improve access to good quality seeds, and promote new varieties for producers.

Finally, it is also important to note the weak development of farmers’ cooperatives. Individual farmers carry out production, storage and marketing. According to Kamdem (2014), collective marketing has a statistically significant and positive effect on the net price received by producers. Accordingly, there is a need to organize and structure this sector to establish efficient farmers’ cooperative networks. Some of these cooperatives could be encouraged to become seed producers based on the experience in Senegal (Clavel & Guèye, 2018). Political will and good policies are also needed to revamp the groundnut value chain in Niger. There are important opportunities for groundnut in Niger given the high demand for cooking oil. In 2011, Niger exported about 160 tons of groundnuts, while in 2013 it imported palm oil valued at $39 million (I.N.S., 2011; Actualitix, 2016). Promoting groundnut processing could help reduce the quantity of cooking oil imports, reduce postharvest losses, and stimulate groundnut production.

## 5. Conclusion

Overall, groundnut has huge potential in Maradi and Zinder regions of Niger. Key challenges include environmental constraints and field and storage pests. These can be addressed through the introduction and dissemination of improved technologies such as drought tolerant and pest resistant varieties, and hermetic bags. Groundnut once made Sahelian farmers prosperous and still has the potential to strengthen economic development and reduce poverty in Niger. Enabling policies that would promote groundnut production, processing and marketing are needed to scale the adoption of proven agricultural technologies and practices.
